# Multicenter prospective study on the burden of rotavirus gastroenteritis in children less than 3 years of age in Spain

**DOI:** 10.1186/s12879-016-1890-7

**Published:** 2016-10-10

**Authors:** J. Arístegui, J. Ferrer, I. Salamanca, E. Garrote, A. Partidas, M. San-Martin, B. San-Jose

**Affiliations:** 1 Unidad de Infectología Pediátrica, Hospital de Basurto, Universidad del País Vasco (UPV/EHU), Avenida de Montevideo 18, 48013 Bilbao, Vizcaya Spain; 2 Pediatría, CAP Roquetes Canteres, Barcelona, Spain; 3 Pediatría, Instituto Hispalense de Pediatría,, Sevilla, Spain; 4 Medical Department. Sanofi Pasteur MSD, Madrid, Spain; 5 OXON Epidemiology, Madrid, Spain

**Keywords:** Gastroenteritis, Rotavirus, Burden, Primary care, Spain

## Abstract

**Background:**

Rotavirus is acknowledged as an important cause of paediatric gastroenteritis worldwide. In Spain, comprehensive data on the burden of rotavirus disease was lacking.

**Methods:**

A prospective, multicenter, observational study was carried out, during the winter season, from October to April 2014 in selected areas of Spain (Catalonia, Basque Country, Andalusia) to estimate the frequency and characteristics of acute gastroenteritis (AGE) and rotavirus gastroenteritis (RVGE) in children ≤3 years of age seeking medical care in primary care and emergency department centres.

**Results:**

Of the 1087 episodes of AGE registered, 33.89 % were RVGE positive. The estimated incidence of RVGE, was 40.3 (95 % CI 36.1–44.8) episodes per 10,000 child-months in children ≤ 3 years of age and the 5-month (December-April) seasonal RVGE incidence rate was 2.01 [1.81–2.24] per 100 children. No vaccination and attending a day care centre were the main risk factors for RV infection. RVGE infected children presented more frequently with fever (63.9 % vs. 45.1 %, *p* = 0.009), vomiting (61.2 % vs. 44.3 %, *p* = 0.015), suffered more dehydration, and were hospitalised and went to the emergency room more often (41.7 % vs. 15.7 %, *p* <0.001) than non-RVGE infected ones. Children were usually more tired (77.5 % vs. 54.2 %, *p* <0.001), tearful, (47.2 % vs. 34.8 %, *p* <0.001), and easily irritated (76.5 % vs. 59.8 %, *p* <0.001), and parents were more concerned (41.7 % vs. 15.7 %, *p* <0.001) and suffered more working rhythm disturbances (39.0 % vs. 22.9 %, *p* <0.001). The cost for families of RVGE cases was significantly higher than the cost of non-RVGE infected ones (47.3 vs 36.7 euros, *p* = 0.011). Vaccinated children suffered less clinical symptoms and no hospitalization. Therefore, vaccination decreases the psychosocial stressors caused by the disease in the family.

**Conclusions:**

Rotavirus infections are responsible for a substantial proportion of AGE cases in children ≤3 years of age in Spain attended at primary care visits. RVGE episodes are associated with greater clinical severity, greater alterations in the child´s behaviour, and higher parental distress. The outcomes of the present study recommend that routine rotavirus vaccination in infants ≤3 years of age could considerably reduce the serious burden of this potentially serious childhood disease.

**Electronic supplementary material:**

The online version of this article (doi:10.1186/s12879-016-1890-7) contains supplementary material, which is available to authorized users.

## Background

Acute gastroenteritis (AGE) is responsible for an important morbidity and mortality in children worldwide. Rotavirus infection is the leading cause of AGE [1] and is more frequently associated with severe presentation and increased hospitalization rates, compared with AGE due to other infectious causes [2]. Rotavirus is highly contagious, and in temperate climates usually peaks in the course of the winter months [3]. Specifically, in Spain, it has been described that the RVGE season occurs between December and April, with the higher incidence observed between January and March [4–6].

The estimated annual rate of clinical rotavirus gastroenteritis (RVGE) is 1 case in every 7 children within the European Union, giving rise to 231 deaths, more than 87,000 hospitalisations, and nearly 700,000 outpatient visits [4]. AGE caused by rotavirus is characterized by watery diarrhoea; with a mean duration of 2–7 days that can lead to severe dehydration requiring hospital treatment and, in some cases, be fatal. It has been estimated that, in Europe, 1 out of 54 RVGE will require hospital admission [7].

RVGE is also related to a significant economic burden for the health care system (medical visits, hospitalizations, treatment costs) and for families (parent work days lost, costs related childcare, etc.) [8, 9]. Taking all these into account, rotavirus infections have been classified as an essential target for vaccination. Moreover, the guidelines published by the European Society for Paediatric Infectious Diseases and the European Society for Paediatric Gastroenterology, Hepatology, and Nutrition [10, 11], as well as by the WHO Strategic Advisory Group of Experts [12] recommend vaccination against rotavirus. Rotavirus vaccines available, a live attenuated monovalent human vaccine (GSK Biologicals) [13] and a live human-bovine reassortant vaccine (Sanofi Pasteur MSD) [14] have demonstrated high efficacy, effectiveness and a good safety profile both in pre-licensure clinical trials and in post-authorization studies [15]. Rotavirus vaccines were introduced in Spain in 2006–2007 but they are not publicly funded by the Spanish National Health Care System although they are recommended by the Spanish Association of Paediatricians and prescribed by paediatricians. Disease burden data generated from epidemiological studies are important for making decisions related to vaccine recommendations at a national level and to enable the monitoring of vaccine impact.

The main objective of the study was to estimate the frequency and characteristics of RVGE and non-RVGE in children from 0 to 3 years of age attended at primary care and emergency department centres in Spain. Other objectives included to determine the clinical symptoms and consequences of both types of GE and to assess the impact of RVGE on affected children and their parents.

## Methods

### Study design

A prospective, multicenter, observational study was carried out during the 2014 winter season (December-April) in 3 specific areas of Spain (Catalonia, Basque Country, Andalusia) to evaluate the frequency of acute gastroenteritis (AGE) and rotavirus gastroenteritis (RVGE) in children from 0 to 3 years of age attended at primary care and emergency department centres.

### Setting

To conduct the study three principal investigators, one for each Autonomous Community, were selected on the basis of their experience as clinical paediatricians and researchers engaged in the management of children with RVGE. These principal investigators were involved in the pre-selection of a convenience sample of a total of 87 paediatricians whose current practice included the management of children with RVGE (24 in Andalusia, 34 in Catalonia, and 29 in the Basque Country), of which only 64 finally participated in the recruitment (15 in Andalusia, 22 in Catalonia, and 27 in the Basque Country). Participant paediatricians, who recruited patients, belong to a total of 33 centres (Andalusia 9, Catalonia 8, and 16 from the Basque Country). Of these, 31 centres and 54 paediatricians were primary care practices, and 2 were emergency services centres, namely the Virgen de Valme Hospital (Andalusia) and the University Hospital of Basurto (Basque Country). The study was carried out based on the 2004 amendment of the Helsinki Declaration, the Guidelines for Good Epidemiological Practice [16], as well as with the local regulatory requirements. The local ethics committee approved the protocol in each area of study (*Comité Ético de Investigación Clínica del l’IDIAP Jordi Gol i Gurina, Comité Ético del Hospital Universitario Virgen del Rocío, Comité de Ética del País Vasco)*

### Type of participants

All children ≤3 years of age, residing within a specific study area during the study period and who were seeking medical intervention for AGE qualified for inclusion in the study. AGE was described as an episode of at least 3 loose or watery stools, and/or vomiting associated with gastroenteritis occurring within a 24-h period in the 7 days prior to the medical visit [17].

Those children who had a previously diagnosed chronic disease of the gastrointestinal tract for which symptoms were compatible with the definition of AGE were not considered eligible for inclusion. In the Basque Country and in accordance with the suggestion of the local ethics committee; children with mucus and bloody stools were also excluded.

Eligible children whose parents did not provide written, informed consent were listed as “not included eligible children”. Data on these children were filed anonymously on a “screening list” that showed only data related to their age and consultation date. This screening list was utilized in the calculations of estimates (incidence).

### Sample size calculations

The sample size was estimated after stratifying by Autonomous Communities (AC). At each AC, the total sample size was calculated on the basis of the number needed to obtain an incidence rate of acute gastrointestinal episodes due to rotavirus between 3.7–9.2 %, as reported in the study REVEAL, with a precision of 95 % and an error of 2–3 % around the estimate. For obtaining this sample, as only 8–12 % gastro-intestinal episodes among children of age corresponding to the study population result in a demand for clinical attention (7.5–10.3 % at primary care paediatricians, 0.2–1.5 % at the emergency room), a total of 350 gastrointestinal episodes per AC was estimated. For obtaining this number of episodes among the study population at each AC, the rate of children of age corresponding to the study population per paediatrician and hospital catchment areas at each AC was estimated in order to include as many paediatricians as needed among the recruiters.

### Data collection

Children who met the inclusion criteria and whose parents signed the informed consent were included consecutively. After informed consent was obtained, the study paediatrician examined each patient and completed a standardized data collection form (DCF) that included the demographic data, environmental factors (breastfeeding, attendance at day care centres, etc), information on clinical symptoms, and illness onset.

One stool sample was collected for rotavirus testing (VIKIA Rota-Adeno immuno-chromatographic test (bioMérieux)® from each child (sensitivity = 96.6 %, specificity = 92.9 %) [18]. If no stool sample was obtained during the baseline visit, parents were requested to collect a stool sample and bring it into the health care center within four days. Children with a positive VIKIA Rota-Adeno immuno-chromatographic test result for rotavirus were classified as RVGE positive (RV+).

To evaluate the characteristics of the episode, parents of each included child were given a diary and were then asked to complete the questionnaire at home until the episode was resolved and to return the completed diary to their paediatrician (Additional file 1: Table S1). During a follow-up visit or phone call, the paediatrician followed-up on whether or not the diary had been completed and returned, and then collected the data in the DCF.

Data collected in the DCF included demographic data (age, gender), environmental factors, date of the AGE onset, rotavirus vaccination, VIKIA Rota-Adeno immuno-chromatographic test result (bioMérieux)® in faeces, clinical presentation of the AGE in the last 24 h (diarrhoea, fever, vomiting, dehydration), consequences of infection (referral of patient to the emergency room and/or hospitalization), treatment received for the infection, AGE symptoms, duration of episode, contact with health services, disease severity (level of dehydration), family members with similar symptoms, work missed by the parents, and additional costs for parents related to the episode. The data collected by researchers in the DCF was integrated into a centralized database that was used for the analysis of results.

### Main variables analyzed

The RVGE episode frequency has been described for the whole sample of patients registered in the study. Monthly incidence rates (IR) per 10,000 children ≤ 3 years of age and 5-month IR per 100 children ≤3 years of age have been calculated from cases for which the investigators’ patient quotas were available (Catalonia and Basque Country).

For the sample of patients who consented to participate in the study, patient characteristics and clinical features have been described including socio-demographic characteristics, environmental factors, clinical data, characterization of AGE, and consequences of the AGE.

## Statistical analyses

### Definition of data sets to be analyzed

**Fig. 1:**
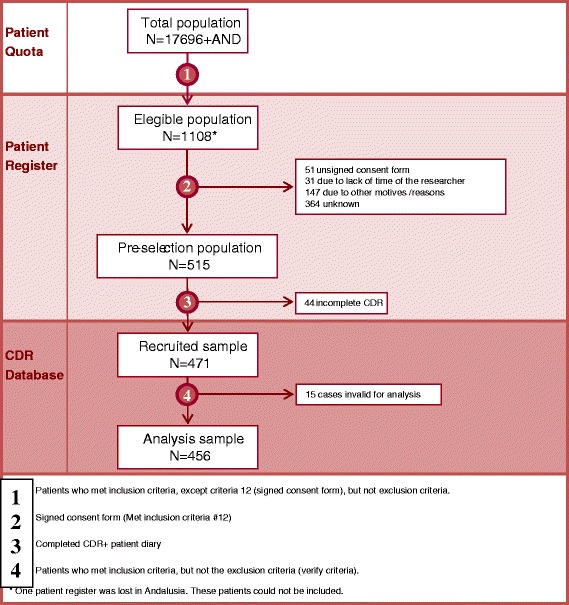
Flow-chart of the study

The reference population used to estimate the incidence values corresponds to the number of children between 0 and 3 years as part of the quotas of the participating researchers (17,696 children). The eligible population is composed of children who suffered an episode of AGE during the study period and who met the inclusion criteria (1,108 children). The preselected sample is the eligible population who gave their informed consent to participate (515 children). The recruited sample consists of subjects that make up the population of pre-selection and that had the DCF and the patient diary filled out (471 patients). The sample per protocol analysis is that part of the preselected sample that effectively meets the inclusion criteria (456 patients) (Fig. 1 and Additional file 2: Figure S1). A description was made and an inferential analysis comparing between confirmed cases of rotavirus infection with other subjects with AGE not caused by rotavirus was performed in this population.

### Statistical methods

Qualitative data were described with frequency tables and percentages; and quantitative data with quantitative descriptive statistics (mean, standard deviation (SD), median, and range values). Incidence has been expressed as monthly incidence rate (per 10,000 children) and a seasonal rotavirus IR (per 100 children). Incidence rates were obtained considering daily RVGE incidence (daily cases of RVGE confirmed/person-time (days) at risk) and the total number of days in the follow-up. Poisson distribution was used to calculate 95 % CI.

The different variables of interest were compared between both groups of study (RVGE+/RVGE-) using the Chi-squared or the Fisher’s exact test for categorical variables, and the Student’s “t” test for independent data or the Mann-Whitney U test in the event that the assumption of normality was not met, to compare quantitative variables. The Kolmogorov-Smirnov test was used to assess if quantitative variables followed a normal distribution. Statistical analyses were performed using SAS software (version 9.2; SAS Institute). Statistical significance was set at *p* < 0.05.

A univariate analysis was performed based on the results obtained from the VIKIA test carried out on the sample population recruited in accordance with the variables included in the prospective study. Then, for those variables where a significant association was detected, a multivariate analysis was carried out to describe the profile differential in those patients with RVGE compared to those with AGE. A logistic regression analysis was used to compare the profiles between RVGE and non-rotavirus AGE patients. The results of the adjustments made to these models have been described using an odds ratio (OR), its 95 % confidence interval (CI) and the corresponding p values obtained.

## Results

During the 5-month study period (December-April), 1,087 episodes of AGE were registered and of them 471 gave their consent to participate in the study. Finally, 456 patients were recruited with a clinical episode of AGE (15 cases were invalid): 107 (23.4 %) from Andalusia, 138 (30.3 %) from Catalonia and 211 (46.3 %) from the Basque Country.

### Frequency and incidence of AGE and RVGE

**Fig. 2:**
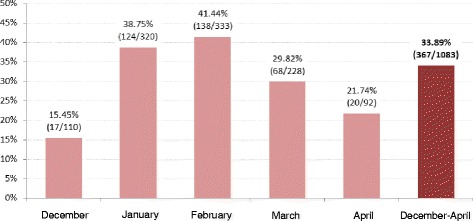
Proportion of acute gastroenteritis attributable to RV from December 2014 to May 2015

Of the 1,087 episodes of AGE registered, 33.89 % were RVGE positive (Fig. 2). The great majority of AGE episodes occurred during the months of January and February (60.3 % of all cases). During these months, the proportion of RVGE cases was 38.75 % and 41.44 %, respectively (Fig. 2).

**Fig. 3:**
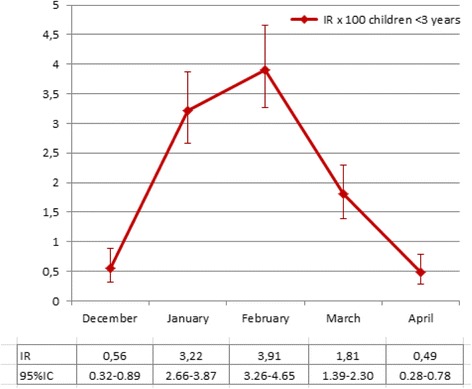
Overall incidence rate of RVGE (Catalonia and Basque Country) from December 2014 to May 2015

Cases from Catalonia and the Basque Country sites were used for incidence calculation (as these investigators’ patient quotas were available). Among the 898 episodes of AGE registered, 38.1 % were positive for RV. The incidence rate of RVGE for the 5-month (December-April) seasonal period was 2.01 [1.81–2.24] per 100 children (Fig. 3). The higher incidence rate of RVGE was registered in February with 3.91 (95 % CI: 3.26–4.65) per 100 children ≤ 3 years of age (Fig. 3).

### Characteristics of the sample

Of the 456 patients with AGE included in the study, 417(91.5 %) were recruited in primary care centres and 39 (8.5 %) in the emergency services, and 201 (44.3 %) patients tested positive for RV in the VIKIA test. The proportion of RV positive cases was 66.7 % (26/39) for cases recruited in the emergency services and 41.9 % (175/417) among primary care cases.

Table 1 shows the epidemiological characteristics of the sample. Mean age of the sample was 16.1 ± 8.9 months. AGE was most prevalent in children of the 6 to 24 month age-group (65.4 % of episodes). Of the 456 patients comprising the total sample, 72.1 % had not been vaccinated against rotavirus, while 24.5 % had a completed vaccination schedule. The children of the sample were mostly breast-fed (83.0 %), but only 13.6 % continued with breastfeeding at the time of the AGE episode. Previous episodes of AGE were reported in 37.9 % of the children and 2.0 % of the children had been previously hospitalised for AGE.

**Table 1:** Epidemiological characteristics of the overall, RV+ and RV- children and possible risk factors of RVGE

		Total *N* = 456 n (%)	RV + *N* = 201 n (%)	RV – *N* = 253 n (%)	*p*-value
Gender	Male	236 (51.9)	95 (47.3)	140 (55.6)	0.079
	Female	219 (48.1)	106 (52.7)	112 (44.4)	
Age-groups (3-groups)	0–12	166 (36.4)	79 (39.3)	87 (34.4)	0.063
	12–24	184 (40.4)	86 (42.8)	97 (38.3)	
	24–36	106 (23.2)	36 (17.9)	69 (27.3)	
Age-groups (5-groups)	0–2	10 (2.2)	4 (2.0)	6 (2.4)	0.028
	2–6	42 (9.2)	14 (7.0)	28 (11.1)	
	6–12	114 (25.0)	61 (30.3)	53 (20.9)	
	12–24	184 (40.4)	86 (42.8)	97 (38.3)	
	24–36	106 (23.2)	36 (17.9)	69 (27.3)	
RV vaccination	Complete	109 (24.5)	20 (10.0)	89 (36.6)	0.001
	Partial	15 (3.4)	2 (1.0)	13 (5.3)	
	None	321 (72.1)	179 (89.1)	141 (58.0)	
Breastfeeding	Yes	376 (83.0)	171 (85.9)	204 (80.6)	0.137
Continue with breastfeeding	Yes	51 (13.6)	30 (17.5)	21 (10.3)	0.041
Number of previous episodes of AGE	1	80 (52.3)	41 (67.2)	39 (42.9)	0.045
	2	42 (27.5)	13 (21.3)	29 (27.5)	
	3	18 (11.8)	5 (8.2)	13 (11.8)	
	>3	13^a^ (8.5)	2 (3.2)	10 (11.0)	
Previous hospitalizations for AGE	Yes	9 (2.0)	3 (1.5)	6 (2.4)	0.737
Attendance at day care center (currently)	Yes	210 (46.6)	80 (40.2)	128 (51.2)	0.020

^a^1 missing data; RV: rotavirus; RV+: VIKIA test positive; RV-: VIKIA test negative; AGE: acute gastroenteritis

### Comparison of RVGE+ versus RVGE-

Tables 1 and 2 show the epidemiological and clinical characteristics of RVGE+ and RVGE- children, respectively. Age distributions of RVGE+ cases were significantly different to RVGE- cases, being 73.1 % and 59.2 % respectively, between 6 and 24 months of age. When comparing the risk factors between RVGE+ and RVGE- patients, statistically significant differences were found in the percentages of vaccination (11.0 % vs. 41.9 %, *p* <0.001), breastfeeding at the time of the episode (17.5 % vs. 10.3 %, *p* = 0.041), number of previous episodes of AGE (*p* = 0.045), and of those currently attending care day centres (40.2 % vs. 51.2 %, *p* = 0.020).

**Table 2:** Clinical characteristics and consequences of AGE overall and in RV positive and RV negative

	Total *N* = 456 n (%)	RV + *N* = 201 n (%)	RV – *N* = 253 n (%)	*p*-value
Symptoms 24 h before the visit				
Fever	229 (50.3)	123 (61.2)	105 (41.7)	0.001
Diarrhoea	424 (98.2)	193 (97.0)	247 (99.2)	0.147
Vomiting	249 (58.2)	132 (69.1)	116 (50.0)	0.001
Dehydration	59 (13.1)	40 (20.3)	19 (7.6)	0.001
Level of dehydration^b^				0.05
A	169 (86.0)	76 (80.9)	84 (91.3)	
B	12 (6.5)	10 (10.6)	2 (2.2)	
C	14 (7.5)	8 (8.5)	6 (6.5)	
Hydration Treatment	255 (60.6)	129 (68.3)	125 (54.3)	0.004
Relatives with similar symptoms	131 (29.3)	63 (32.1)	68 (27.3)	0.267
VIKIA test (at the visit)	411 (93.2)	180 (93.3)	229 (93.1)	0.943
Patient referral	19 (4.2)	8 (4.0)	11 (4.4)	0.837
Emergency department	6 (31.6)	1 (12.2)	5 (45.5)	0.177
Hospital		7 (87.5)	6 (54.5)	
Symptoms and severity of the current AGE episode				
Fever	224 (54.1)	110 (60.1)	114 (49.8)	0.03
Diarrhoea	442 (96.9)	197 (98.0)	243 (96.0)	0.230
Vomiting	249 (55.6)	130 (65.0)	118 (48.0)	0.001
Level of dehydration^b^				0.051
A	302 (93.2)	138 (90.2)	162 (95.9)	
B	8 (2.5)	7 (4.6)	1 (0.6)	
C	14 (4.3)	8 (5.2)	6 (3.6)	
Hospitalization	15 (5.0)^a^	10 (6.7)^a^	5 (3.3)^a^	0.007
Number of visits to the emergency department				0.006
1	93 (25.9)	55 (33.1)	38 (19.8)	
2	21 (5.8)	11 (6.6)	9 (4.7)	
≥ 3	4 (1.1)	3 (1.8)	1 (0.5)	
Consequences of AGE				
New relatives infected	157 (35.2)	95 (48.0)	62 (25.1)	0.001
Child cries more than usually	276 (61.3)	147 (73.5)	128 (51.4)	0.001
Child is distressed	303 (67.3)	153 (76.5)	149 (59.8)	0.001
Child plays less than usually	264 (59.1)	143 (71.9)	120 (48.6)	0.001
Child is more tired than usually	291 (64.7)	155 (77.5)	135 (54.2)	0.001
Level of worry of parents, mean (SD)	6.7 (2.29)	7.1 (2.10)	6.4 (2.38)	0.001
Sleep disturbances	394 (87.0)	182 (90.5)	210 (84.0)	0.040
Need of external aid	164 (36.4)	88 (44.2)	75 (30.1)	0.002
Alteration of working rhythm	135 (29.9)	78 (39.0)	57 (22.9)	0.001
Having loss of working days	95 (21.0)	50 (24.9)	45 (18.0)	0.075
Having income loss	63 (14.1)	32 (16.1)	31 (12.6)	0.295
Income loss, (euros), mean (SD)	115.1 (96.96)	141.6 (113.76)	89.5 (70.39)	0.108
Costs, (euros), mean (SD)	41.6 (33.6)	47.3 (38.2)	36.7 (28.3)	0.011

*SD* standard deviation

^a^some missing data (*n* = 300)

^b^Level of dehydration: A = No dehydration, B = some dehydration, C = severe dehydration

During the previous 24 h prior to paediatrician visit, RV infected patients presented a higher frequency of fever (61.2 % vs. 41.7 %, p <0.001) and vomiting (69.1 % vs. 50.0 %, *p* <0.001), and the number of vomiting episodes was also higher (3.0 [IQR: 2.0–5.0] vs. 2.0 [IQR: 1.0–3.0], *p* = 0.004). Moreover, RVGE+ patients had a higher incidence of dehydration (20.3 % vs. 7.6 %, *p* <0.001) and the severity of this dehydration was also higher. RVGE+ cases needed hydration treatment more frequently (68.3 % vs. 54.3 %, *p* = 0.004) than RVGE- ones.

During the whole course of the episode, RVGE+ children presented more frequently with fever (60.1 % vs. 49.8 %, *p* = 0.03) and vomiting (65.0 % vs. 48.0 %, *p* = 0.001), and required a higher number of visits to the emergency room (41.5 % vs. 25.0 %, *p* <0.001) than those RVGE- ones. Hospital admission was also more frequent among RVGE+ than RVGE- children (6.7 % vs. 3.3 %, *p* = 0.007) (Table 2).

In families where the children were infected with RV, the percentage of new infections among relatives was higher (48.0 % vs. 25.1 %, *p* = 0.001).

With regards to the impact of the GE episode on children, those with RV infection cried more (73.5 % vs. 51.4 %, *p* <0.001), were more irritable (76.5 % vs. 59.8 % *p* <0.001), played less (71.9 %% vs. 48.6 %, *p* < 0.001), and were more tired than usual (77.5 % vs. 54.2 %, *p* <0.001) than non-RV infected children.

Parents whose children had a RV infection were more concerned (10-points visual scale punctuation of 7.0 [IQR: 6.0–9.0] vs. 6.0 [IQR: 5.0–8.0], *p* = 0.001) and reported more sleep disturbances (90.5 % vs. 84.0 %, *p* = 0.040) than those of the non-RV infected children. As a result, these parents have needed more external support (44.2 % vs. 30.1 %, *p* = 0.002) and more modifications to their working schedules (39.0 % vs. 22.9 %, *p* <0.001) than those parents of non-RV infected children. The additional mean cost related to the GE episode was also higher in families whose children suffered a RV infection (€ 47.3 [38.2] vs. € 36.7 [28.3], *p* = 0.011) than those whose children were not RV infected (Table 2).

In the multivariate analysis, significant differences in RV vaccination, fever, episodes of vomiting in the previous 24 h, and child´s behaviour (more tired) were obtained (Table 3).

**Table 3:** Epidemiological and clinical differences between children with (VIKIA+) and without rotavirus. Univariate and Multivariate analysis

		Univariate analysis				Multivariate analysis (*N* = 375)		
		*N*	OR	IC 95 %	*p*-value	OR	CI 95 %	*p*-value
Gender	Male	453			0.080			
	Female		1.395	0.961–2.023	0.080			
Age (months)		454	0.978	0.957–0.999	0.038	0.984	0.958–1.010	0.215
RV Vaccination	Complete vaccination	444			<0.001			<0.001
	Partial vaccination		0.685	0.143–3.278	0.636	0.751	0.144–3.919	0.735
	No vaccination		5.649	3.316–9.625	<0.001	5.758	3.196–10.374	<0.001
Breastfeeding	No	452			0.138			
	Yes		1.467	0.884–2.435	0.138			
Attendance at day care centre	No	449			0.021			
	Yes		0.641	0.440–0.934	0.021			
Fever 24 h. befote the visit	No	453			<0.001			0.018
	Yes		2.208	1.512–3.223	<0.001	1.779	1.103–2.868	0.018
Number of episodes of vomiting 24 h. before the visit		423	1.246	1.138–1.363	<0.001	1.112	1.000–1.236	0.050
Number of depositions 24 h. before the visit		448	1.004	0.953–1.059	0.873			
Dehydration 24 h. before the visit	No	448			<0.001			
	Yes		3.111	1.737–5.569	<0.001			
Rehydratation treatment 24 h. before the visit	No	419			0.004			0.170
	Yes		1.806	1.209–2.698	0.004	1.413	0.862–2.316	0.170
Fever returning at the follow-up	No	412			0.037			
	Yes		1.520	1.026–2.252	0.037			
Number of daily episodes of vomiting during the follow-up		446	1.219	1.111–1.338	<0.001			
Maximum number of daily depositions during the follow-up		454	1.085	1.024–1.149	0.005			
Grade of dehydration during the follow-up	A	322			0.110			
	B		8.207	0.998–67.456	0.050			
	C		1.565	0.530–4.621	0.417			
Hospitalization	No	449			0.108			
	Yes		2.094	0.850–5.157	0.108			
Visits general practitioner/paediatrician	No	429			0.347			
	Yes		1.466	0.660–3.255	0.347			
Visits to emergency department	No	358			<0.001			
	Yes		2.134	1.361–3.345	<0.001			
Phone consultations	No	337			0.231			
	Yes		1.335	0.832–2.142	0.231			
Child´s behaviour: Cries more	No	449			<0.001			
	Yes		2.621	1.756–3.912	<0.001			
Child´s behaviour: is more irritable	No	449			<0.001			
	Yes		2.184	1.445–3.303	<0.001			
Child´s behaviour: Plays less	No	446			<0.001			
	Yes		2.702	1.816–4.019	<0.001			
Child´s behaviour: Is more tired	No	449			<0.001			0.002
	Yes		2.909	1.921–4.405	<0.001	2.277	1.357–3.819	0.002
Impact on the parents: worried because of the symptoms	No	451			0.092			
	Yes		3.038	0.836–11.040	0.092			
Impact on the parents: Sleep disturbance	No	451			0.043			
	Yes		1.824	1.020–3.262	0.043			
Impact on the parents: Need of external aid	No	448			0.002			
	Yes		1.839	1.246–2.715	0.002			
Impact on the parents: Alteration of the working rhythm	No	449			<0.001			0.264
	Yes		2.154	1.429–3.245	<0.001	1.351	0.797–2.290	0.264
Impact on the parents: Loss of working days	No	451			0.076			
	Yes		1.508	0.958–2.376	0.076			

*OR* Odds Ratio, *CI 95 %* 95 % Confidence interval

Dependent variable of logistic regression: Result of the VIKIA test for RV (Positive = 1/Negative = 0)

## Discussion

This is the first multicentre study to assess the incidence and burden of RVGE among children ≤ 3 years of age in outpatient settings carried out in Spain. Our findings highlight the burden and characteristics of RVGE in different areas of the country. The overall incidence rate of RVGE was found to be 2.01 per 100 among children less than 3 years of age, with a seasonal peak of 3.91 per 100, with RV infection accounting for 33.89 % of acute GE episodes in these settings. This proportion is similar to those seen in previous European studies involving primary care settings [6, 17, 19] and showed RV to account for a third of primary care consultations for AGE among children. In Spain, previous studies [8, 20] carried out during 2005–2007 reported a lower proportion (13–15 %) of AGE attributable to RV among children examined by primary care physicians. However, the difference in the burden of RV disease is known to vary over time and many factors (age of the children recruited, methods of diagnosis of RVGE, etc.) can influence these figures. The 5-months seasonal incidence rate is in accordance with previous estimates for Spain obtained in the REVEAL study, which reported an annual incidence rate of 4.73 % in children < 5 years of age.

The proportion of AGE attributable to RV varied by age and was highest in children 6–24 months of age (73.1 %). Moreover, AGE attributable to RV affects children of younger ages than AGE not associated to the RV, with the subsequent clinical repercussions. This is consistent with the results found in similar studies throughout the world [9, 21–23]. Furthermore, this can be explained by the fact that at < 6 months of age maternal antibodies have a protective effect, while at >24 months children may have already developed a natural immunity due to recurrent rotavirus infections. In this study, the peak age of 12–24 months is consistent with a European study showing the peak of infection occurring in the second year of life [24]. As opposed to what happens in under-developed areas where the peak occurs between 6–12 months [25]. The early peak of RVGE in these countries may result from early exposure to contaminated sources as well as over-crowded homes [26]. In this study, the peak incidence of RVGE occurred in January-February, differing slightly from the seasonal distribution described for Spain in the REVEAL study in which the peak incidence of RVGE was December-January [6] and was probably related to the lower recruitment rate at the beginning of the study.

With regards to environmental factors, it is observed that only half of children with AGE episodes attended day care centres (40.2 % of the RVGE+ and 51.2 % of the RVGE- children), which contrasts with the hypothesis that attending these centres may be a risk factor for RVGE infection [27].

Rotavirus GE cases were related with a higher presence and severity of symptoms (presence of fever, vomiting, dehydration) that non-RVGE [9, 28, 29]. These more severe consequences (mainly dehydration), implies a more frequent use of health care resources (physicians and emergency room visits and hospitalizations) for RV infection. Although, the emergency services were underrepresented in our study, the proportions of RVGE+ cases in this setting was considerably higher (66.7 % vs 41.9 %) compared to the those of the primary care centres. This observation is consistent with findings from previous studies [30].

A recent review estimated that RVGE costs the Spanish national health system EUR 28 million a year, has a considerable psychosocial impact on the family, and causes productivity loss in two-thirds of parents (mean of 4 days) [9]. The results of our study showed that children infected with GE caused by RV presented with the greatest number of infections. Also, it showed the negative effects to the overall general condition of the children (cries more, played less, is more irritable, more tired than usual) and to that of the parents (more concerned, greater need of external help, and a greater number of disturbances to the working rhythm); results similar to those obtained in other studies [28, 31]. Probably, this higher impact on parents in the cases of RVGE+ children is related to the more severe symptoms observed in RV infected children and the higher number of house members infected compared to RVGE- cases. With regards to the loss of working days and loss of income, unlike those reported in other studies [9]; our study showed no significant differences although an increase in the additional costs related to the AGE episode was observed.

Previous studies suggested that implementing universal vaccination could have an impact on hospital admissions, emergency and paediatric visits related to RVGE [9]. Our study results were in line with these observations. These findings show evidence on the impact of vaccination in reducing hospitalisations [32, 33]. They also provide strong support for the theory that the vaccination of infants against RV may have a major impact in reducing RVGE morbidity, as well as, in alleviating the pressure on healthcare services due to AGE among young children in Europe. Note that other European studies suggest a more than 80 % reduction of hospitalizations caused by Rotavirus in such cases where vaccination rates for this disease are high. Finally, rotavirus gastroenteritis has a considerable psychosocial impact on the family and causes high levels of stress among parents [34] and the results of the study suggest that the burden of RVGE on children and their families could be substantially reduced by the routine rotavirus vaccination of infants. Moreover, some studies suggest that RVGE is a common infection in family members, parents and caretakers of RV infected children [35–37] and a significant deficit in diarrheal cases in older children and adults has been related to an additional indirect effect of vaccination [38]. Further investigations are needed to analyze the cost-effectiveness of RV vaccination among children less than 3 years of age.

### Strengths and limitations

The limitations of the study are those of any observational study. Only those children that were seeking health care were included in the study, but looking at the characteristics of the sample it is reasonable to assume that non-included cases would have had similar traits [5]. The recruitment rate at the beginning of the study was low (1 per 5 attended children), but this was later increased. Other limitations were that the patient quota of participating researchers in the private centres of Andalusia could not be reached and this fact limited the overall incidence rate estimation for this autonomic community and that the emergency services were underrepresented. Finally, we limited the period of the study to the season for RVGE that extended from December to April, since assessing rotavirus seasonality was not the objective of the study. Nevertheless, the use of a common protocol consisting of the standard data collection by the general practitioners in the different geographical areas, the systematic evaluation of consecutive AGE patients from the different centres, and the laboratory confirmation of RVGE strengthened the results.

## Conclusions

In summary, rotavirus infections make up for a substantial proportion of AGE cases in children ≤ 3 years of age in Spain attended at primary care consultations. RVGE as compared with AGE due to other infectious causes is more frequently associated with severe symptoms and increased hospitalizations,. The severity of symptoms may cause anxiety, sleep, and working rhythm disturbances in the affected child’s parents, as well as discomfort to the child. The routine rotavirus vaccination of infants ≤3 years of age could considerably reduce the significant burden of this potentially serious childhood disease.

## Additional files

Additional file 1: Table S1. Parent’s questionnaire. This file is the questionnaire administered to each child´s parent. (DOC 33 kb)
Additional file 2: Figure S1. Flow-chart of the study considering each autonomous community. This figure represents the flow-chart of the study detailed by the three 3 specific geographical areas of Spain analyzed (Catalonia, Basque Country, Andalusia). (DOC 128 kb)

## Abbreviations

AC: Autonomous communities
AGE: Acute gastroenteritis
GE: Gastroenteritis
IQR: Interquartile range
IR: Incidence rate
OR: Odds ratio
RV: Rotavirus
RVGE: Rotavirus gastroenteritis
SD: Standard deviation.
