# Supplementary material for: Multicenter prospective study on the burden of rotavirus gastroenteritis in children less than 3 years of age in Spain
Source: BMC Infect Dis. 2016 Oct 10;16:549. doi: 10.1186/s12879-016-1890-7 (PMC5057213; doi:10.1186/s12879-016-1890-7)
Supplement: Additional file 1: Table S1. — Parent’s questionnaire. This file is the questionnaire administered to each child´s parent. (DOC 33 kb) [file 12879_2016_1890_MOESM1_ESM.doc]

## Parents Questionnaire

## 1. Conclusion of symptoms (dd/mm/yy): / / /

2. Symptoms and gastroenteritis severity:

| **Parameter** | **Results** |
| --- | --- |
| Maximum number of depositions per day  Diarrhoea duration  Maximum number of vomiting episodes per day  Duration of vomiting  Maximum registered temperature  Dehydration (%)*  Treatment (Rehydratation/ Hospitalization) | □□ Depositions  □□ Days  □□ Episodes  □□ Days  □□ ºC  □ A □ B □ C  □ R □ H |

*Dehydratation: A) Treated at home; B) Oral rehydration at the hospital; C) IV therapy at the hospital.

3. Has the child required hospitalization? □ Yes □ No

If affirmative, number of days □□

4. Child´s behaviour:

Cries more than usual □ Yes □ No

Irritable □ Yes □ No

Less playful □ Yes □ No

More tired than usual □ Yes □ No

5. Impact on parents:

For the two first items, indicate the adequate value of its impact on parents, with 10 being the value representing the greatest impact:

☺--/----/----/----/----/----/----/----/----/----/--☻

1 2 3 4 5 6 7 8 9 10

Worry about the symptoms □ Yes □ No Value:__ __

Sleeping disturbances □ Yes □ No Value:__ __

Need for external aids □ Yes □ No

Changes of working rhythm □ Yes □ No

Loss of working days □ Yes □ No

Financial difficulties: loss of income □ Yes □ No

Financial difficulties: added expenses* □ Yes □ No

Examples: extra diapers, treatments, caregivers,…

In the case of loss of working days, how many working days were lost? __ __ days

In the case of loss of income or added expenses, how much money do you estimate?

Loss _______ euros Expenses _______ euros

In the case where the child was NOT hospitalized, indicate the number of visits and/or telephone calls to the health services during the acute gastroenteritis episode:

Visits to the general practitioner/paediatrician __ __ times

Visits to the emergency department __ __ times

Medical telephone calls __ __ times

Has been any family member infected since the last visit? □ Yes □ No

Number of family members __ __
