# Supplementary material for: Multicenter prospective study on the burden of rotavirus gastroenteritis in children less than 3 years of age in Spain
Source: BMC Infect Dis. 2016 Oct 10;16:549. doi: 10.1186/s12879-016-1890-7 (PMC5057213; doi:10.1186/s12879-016-1890-7)
Supplement: Additional file 2: Figure S1. — Flow-chart of the study considering each autonomous community. This figure represents the flow-chart of the study detailed by the three 3 specific geographical areas of Spain analyzed (Catalonia, Basque Country, Andalusia). (DOC 128 kb) [file 12879_2016_1890_MOESM2_ESM.doc]

Total population

N=¿?

**Andalusia**

Total population

N=5557

Total population

N= 12139

Elegible population

N=194*

Elegible population

N=369

Elegible population

N=545

Pre-selection population

N=118

Pre-selection population

N=143

Pre-selection population

N=254

Recruited sample

N=114

Recruited sample

N=143

Recruited sample

N=214

Analysis sample

N=107

Analysis sample

N=138

Analysis sample

N=211

9 no consent

1 due to lack of time of the researcher

37 due to other motives /reasons

29 unknown

22 no consent

8 due to lack of time of the researcher

67 due to other motives /reasons

129 unknown

4 incomplete CDR

7 invalid cases

22 no consent

22 due to lack of time of the researcher

43 due to other reasons

204 unknown

40 incomplete CDR

5 invalid cases

3 invalid cases

**Patient**

**Quota**

**Patient Register**

**CDR Database**

Patients who met inclusion criteria, except criteria 12 (signed consent form), but not exclusion criteria.

Signed consent form (Met inclusion criteria #12)

Completed CDR+ patient diary

Patients who met inclusion criteria, but not the exclusion criteria (verify criteria).

* One patient register was lost in Andalusia. These patients could not be included.

**Basque Country**

**Catalonia**
